# Case studies on IoT monitoring of debris flow and landslides

**DOI:** 10.1371/journal.pone.0361584

**Published:** 2026-11-04

**Authors:** Lei Huang, Yung-Ming Cheng, Shu-min Wei, Miao-miao Gie

**Affiliations:** 1 College of Architecture and Civil Engineering, Sanming University, Sanming, China; 2 Department of Civil Engineering, The University of Hong Kong, Hong Kong, Hong Kong Special Administrative Region, China; 3 Shenzhen Antai Data Monitoring Technology Company Limited, Shenzhen, China; 4 College of Architectural Engineering, Taishan College of Science and Technology, Taishan, China; 5 College of Civil Engineering and Architecture, Wenzhou University, Wenzhou, China; Apeejay Stya University, INDIA

## Abstract

Aiming at the critical demand for real-time monitoring of slope disasters in mountainous and urbanized regions, this study develops Internet of Things (IoT)-based real-time monitoring systems to resolve the issues of data delay and fragmentation in conventional manual monitoring. Through three engineering cases conducted by the authors, we demonstrate the adaptability of IoT technology in slope disaster monitoring under different instability mechanisms (debris flows and landslides) and geological conditions (artificial-reinforced and semi-natural slopes). Specifically, in Case 1, a novel IoT monitoring system for debris flows integrated with the structural characteristics of rigid barriers is proposed. In Cases 2 and 3, multi-type monitoring sensors and equipment are deployed, including deformation sensors, rainfall gauges and video surveillance devices, to achieve accurate early warnings of rainfall-induced landslides. The novelty of this work lies in proposing a unified IoT architecture scheme applicable to different types of slope disasters. This scheme systematically integrates physico-mechanical sensing devices, a solar-battery collaborative power supply system, video monitoring equipment, data transmission methods, early warning modes, and a cloud-based processing module. Furthermore, the effectiveness of the proposed scheme is validated via successful early warning cases, and this scheme provides an effective technical framework for slope disaster prevention and control.

## Introduction

Slope hazards, such as debris flows and landslides, pose significant threats to human lives, infrastructure, and economic activities in mountainous and urbanized regions. Stability assessment and supporting measures are critical for disaster prevention. While stability evaluations and engineering designs (e.g., rigid/flexible barriers and retaining walls) are critical for mitigating risks, inherent uncertainties in geotechnical engineering inevitably exist in the practice of slope risk assessment [1–11].This causes deviations and inaccurate estimates in slope risk assessment. Therefore, it is crucial to monitor the actual state of slopes. Traditionally manual monitoring methods, which rely on periodic field measurements, suffer from delayed data acquisition, human resource constraints, and an inability to capture rapid slope dynamics during emergencies. These shortcomings highlight the urgent need for advanced monitoring systems capable of real-time data collection, automated analysis, and immediate hazard warnings.

Recent advancements in the Internet of Things (IoT) have enabled transformative progress in slope disaster monitoring. Existing studies have explored IoT systems integrating sensors with cloud computing for real-time data processing and early warning [12–21]. For debris flow monitoring, systems should employ impact and distance sensors to track debris dynamics, while landslide monitoring should focus on structural displacement and environmental parameters. Currently, there are relatively few studies and practical applications on monitoring debris flow in the context of IoT. Meanwhile, existing research has not yet conducted a detailed discussion on the differences between the IoT monitoring systems of debris flow and landslides. Considering the diversity of slope instability forms, the key technical points for constructing IoT slope monitoring systems still need to be summarized and generalized through more practical engineering cases.

This paper systematically fulfills the critical demand for summarizing the core technical points of IoT-based slope disaster monitoring systems by presenting three representative engineering cases covering diverse slope instability scenarios. The case studies collectively demonstrate the adaptability of IoT systems to varying failure mechanisms (debris flows and landslides), geological settings (reinforced and semi-natural slopes), and triggering factors (typhoon-induced rainfall). By combining field deployments, technical design details, and post-event analysis, this study demonstrates how IoT technology can overcome limitations in traditional monitoring methods, such as delayed responses and limited data integration. The innovation of this paper lies in the proposal of an IoT debris flow monitoring scheme integrating rigid debris flow barriers and intelligent monitoring systems, which has not been reported in existing literature. More importantly, the core foundational innovation of this work lies in the proposal of a unified IoT architecture scheme applicable to different slope disaster types, which systematically integrates full-chain technical modules, including physico-mechanical sensing devices, a solar-battery collaborative power supply system, video monitoring equipment, data transmission approaches, early warning modes, and a cloud-based processing module. Meanwhile, the reliability and field effectiveness of the proposed architecture are verified through successful real early warning cases.

### Case 1: IoT monitoring system for debris flow

When conducting debris flow monitoring in a traditional way, technicians are required to obtain field measurement results. However, there are many cases where such manual monitoring is too late to collect the necessary information. With the advancement of information technology and mobile communication, there is an increasing trend to use IoT in the construction field. Considering the nature of debris flow problems, it is crucial to precisely predict the initiation of failure, the flow of debris, and the consequences of debris flows. Therefore, monitoring the debris flow process is an important issue. Wrachien and Brebbia [22] have provided some guidelines for such monitoring work, but so far, there seems to be no universal guideline. The results of field measurements can also provide data for theoretical advancement. While the traditional monitoring approach is more passive and delayed, using IoT with an immediate response is the current trend in smart monitoring. Thus, it is necessary to address how to construct an IoT monitoring system along with a classical barrier structure.

To safeguard against the debris flow problem, both rigid and flexible barriers are often used. The design of rigid and flexible barriers is covered by Kwan and Koo [23] and Sze et al. [24]. Laboratory and field tests have been conducted by Sun and Law [25], Cheng et al. [26], and Koo et al. [27]. The mechanism for debris flow and the modeling have been studied by VanDine [28], Cheng et al. [26], Chen [29], Wrachien and Brebbia [22], Takahashi [30], and Wu [31]. In general, if the potential dangerous zone is near premises, the use of rigid barriers is more common. By contrast, the flexible barriers have the advantages of fast construction and low cost, which is suitable for protecting a large area. Given the actual situation of our project, the rigid barrier is adopted to establish the prevention structure in the current work.

It should be noted that no permits were required for the field monitoring work in this study. The research areas are not protected areas for wild animals and plants. They are also not regions where scientific research is prohibited by government agencies. In addition, this study focuses on slope disaster prevention and serves livelihoods for residents. It does not involve any restricted operations or protected ecological resources.

### Overview of the IoT monitoring system for debris flows

In this section, a rigid-barrier monitoring project conducted by the authors is presented with a novel design methodology. The site is located on the hillside about 100 m to the northeast of Hang Tsuen, New Tung Chung, Hong Kong Special Administrative Region, China (Fig 1). The rigid retaining-wall facade is 19.5 m long and 4.5 m high, which is approximately trapezoidal in shape. The monitored retaining wall is a reinforced concrete wall with mini piles and soil nails. The monitoring system of this project consists of two parts: the acquisition end and the cloud data-processing system. The design framework of this system is described as follows and shown in Fig 2.

**Fig 1:**
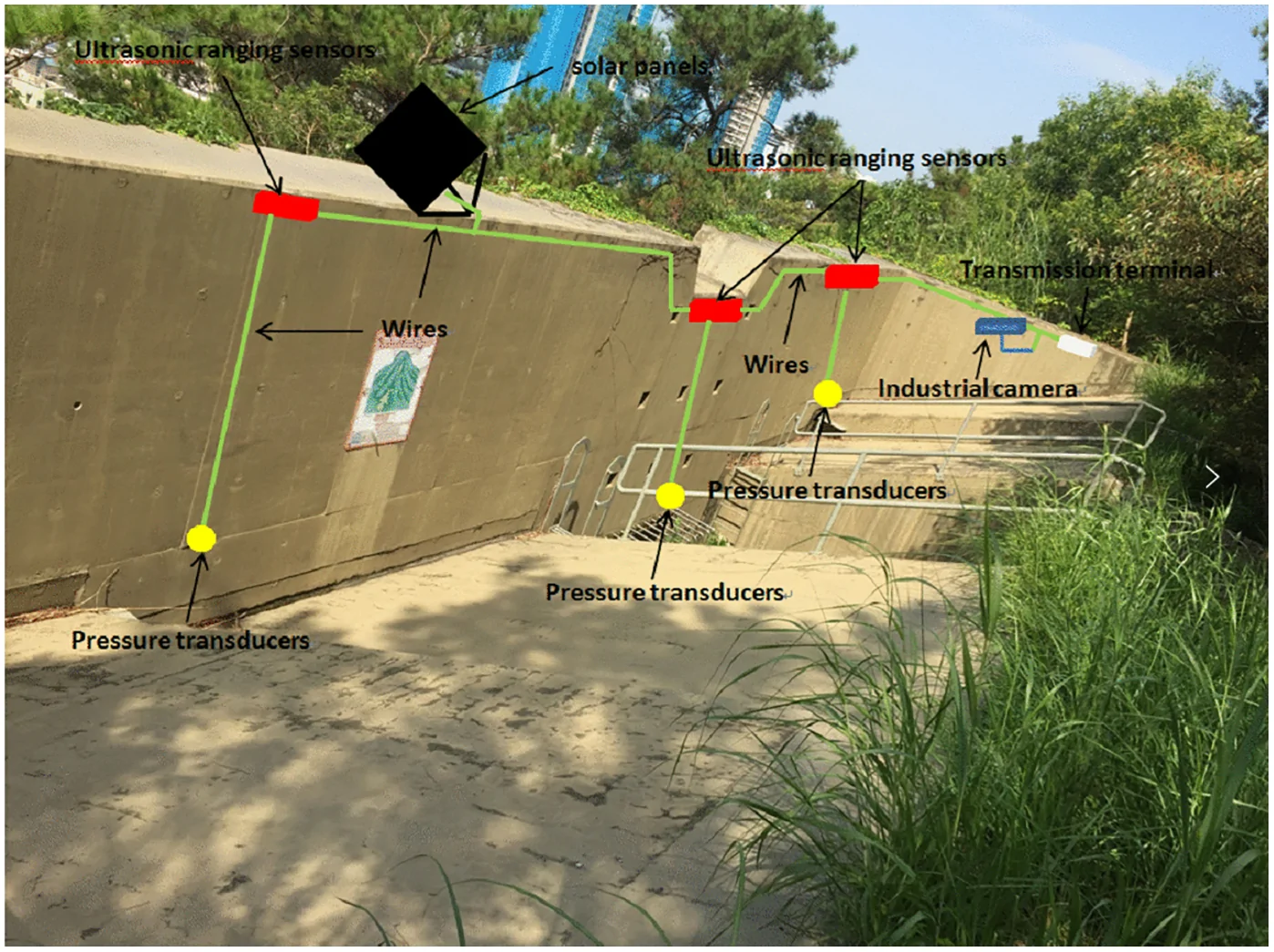
The IoT monitoring system for debris flow (Republished from Shenzhen Antai Data Monitoring Technology Co., Ltd. under a CC BY license, with permission from Shenzhen Antai Data Monitoring Technology Co., Ltd., original copyright 2023).

**Fig 2:**
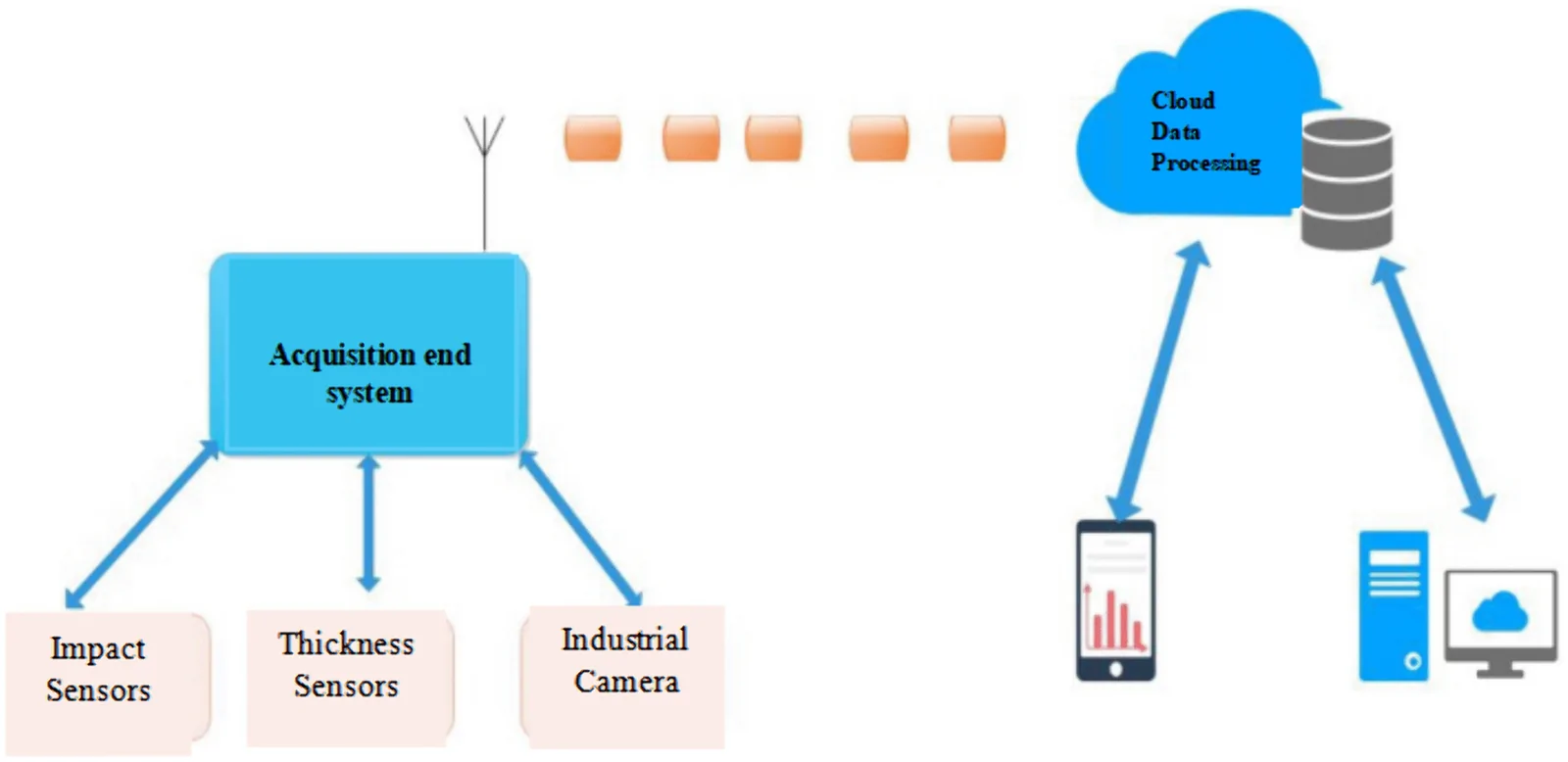
The framework of the IoT Monitoring system.

(1) Acquisition end system

The acquisition end system is composed of pressure sensors, distance sensors, an image acquisition sensor (i.e., industrial camera in Fig 1) and a data transmission module (i.e., transmission terminal). The pressure and distance sensors collect quantitative data, while the industrial camera is used to collect images. Subsequently, the collected data are transmitted to the cloud data processing center through the data transmission module.

(2) Cloud data processing system

After receiving the data transmitted by the acquisition end, real-time processing will be performed on the measured data and images in the cloud data processing system. Subsequently, the monitoring report will be generated, which will be sent to the specified user.

In general, the design concept of this system is that when a debris flow disaster occurs, the system can accurately detect the distance between the debris flow mass and the retaining wall in real time and send out an alarm signal to users immediately. This signal will serve as a crucial basis for the evacuation of surrounding residents. When the debris flow impacts the retaining wall, the pressure sensor will be activated and accurately record the impact pressure of the debris flow. These pressure data will serve as important parameters for academic research and the formulation of relevant specifications. In the meantime, the high-frequency shooting mode of the industrial camera will be activated to record the dynamic trajectory and morphology of the debris flow in a coordinated manner. These generated images are important reference materials for future academic studies. This system not only ensures the effectiveness of real-time early warning, but also plays a vital role in the data collection of debris flow site research, aiming to provide all-around support for debris flow prevention and control work. Taking energy consumption into account, in order to achieve the above functions, the system has the following two detection modes:

- Surveillance Mode. Surveillance mode is in effect during periods when the debris impact sensors have not been triggered for unscheduled readings by a debris impact incident. Under such conditions, the system shall collect data (e.g., debris thickness, stress level, images, battery power level, and temperature) every four hours.
- Detection Mode. The detection mode is associated with specific periods. It is activated when debris impact sensors are triggered by a debris impact incident, prompting unscheduled readings. In such cases, the system will promptly collect time-stamped measurements of the stress level and debris thickness at the moment of impact. After the detection mode is activated, additional readings will be obtained at intervals of no more than two minutes for a duration of one hour. Once this one-hour period elapses, the system will switch back to the surveillance mode for continuous monitoring. This detection mode is initiated each time the sensor detects a debris impact event. Moreover, the activation of the detection mode will trigger the industrial cameras to start collecting photographic images.

### Acquisition end system

In the acquisition end system, several types of sensors are adopted, as shown in Fig 3. The sensors and auxiliary equipment are described as follows:

- Debris flow impact sensor: Three pressure sensors are installed in the acquisition end. Table 1 presents the technical parameters for the pressure sensors. In addition, there is a sub-transmission terminal for each pressure sensor, and the battery is connected through a set of wires to the sub-terminal. The sub-terminal will then be connected to the sensors through another set of wires which allow both electric supply and data transmission.
- Distance measurement sensor: Three ultrasonic distance sensors are installed. Table 2 presents the technical parameters for the ultrasonic distance sensors. The measurement data are transmitted through wired output. There is a sub-transmission terminal for each ultrasonic ranging sensor, and the battery is connected through a set of wires to the sub-terminal. The sub-terminal will then be connected to the sensors through another set of wires which allow both electric supply and data transmission.
- Industrial camera: An industrial camera is installed. The industrial camera can take images every 4 hours even when there is no collision to trigger the detection mode. Additionally, it supports remote adjustment of the monitoring frequency. The industrial camera is connected to the data acquisition and transmission terminal gateway to capture and upload the site photos to the online system. In addition, it can take photos in low illumination (moonlight level), and even in dark environments.
- Data acquisition and transmission terminal gateway (Fig 4): This equipment collects the data of the above sensors through sub-transmission terminals and uploads them to the cloud system through 4G wireless transmission. This transmission terminal can store and process data for wireless transmission. In addition, it has the function of local data storage in a secure digital (SD) card inside the terminal. Moreover, if the data fail to be sent due to an interruption, continuous transmission functionality can be enabled..
- Power supply device: Solar power supply mode is adopted in the system (Fig 5). The basic configuration is: solar panel, colloidal energy storage battery, and solar controller. It can guarantee the normal operation of sensors and gateway system for at least three years. When the power supply is interrupted, the battery can sustain the system for at least one month when fully charged.
- Waterproofing: Each device is equipped with waterproofing measures to protect it from damage caused by precipitation and other factors in the field environment.
- Equipment management system: The equipment management system will monitor the running status of the equipment. When the equipment fails, it will promptly push the equipment failure information to relevant personnel and remind them to repair and replace the equipment. Relevant personnel will regularly inspect the monitoring site and carry out performance tests on the monitoring equipment to ensure the normal operation of the system.

**Fig 3:**
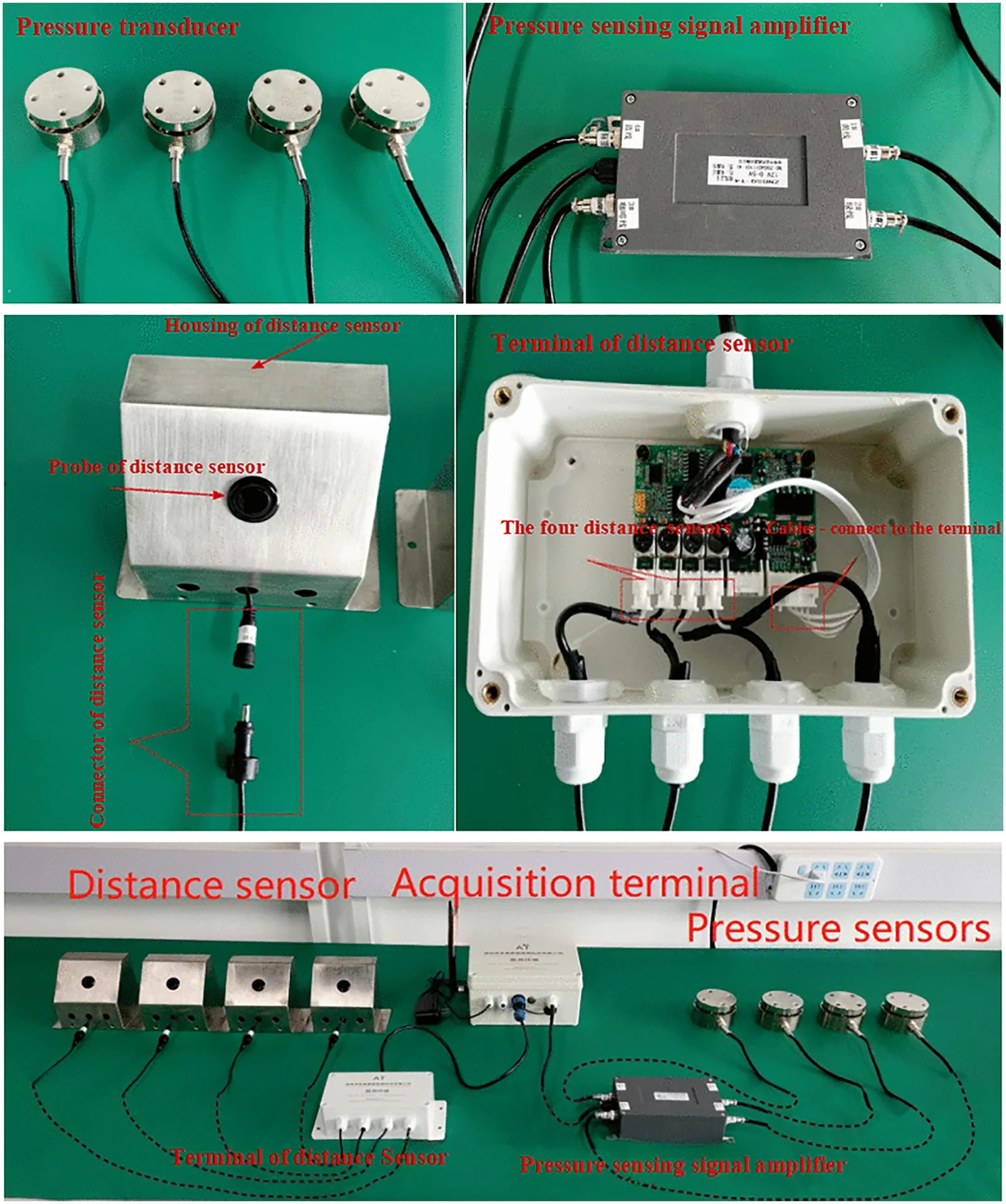
Sensors and aided-equipment used in Case 1 (Republished from Shenzhen Antai Data Monitoring Technology Co., Ltd. under a CC BY license, with permission from Shenzhen Antai Data Monitoring Technology Co., Ltd., original copyright 2020). (a) Pressure sensors. (b) Distance sensors. (c) Connection details for the sensors.

**Table 1:** Technical parameters for the pressure sensor.

Parameter	Unit	Technical Indicators	Parameter	Unit	Technical Indicators
Sensitivity	mV/V	2.0 ± 0.05	Sensitivity temperature	≤%F·S /10℃	± 0.03
Non-linearity	≤%F·S	± 0.03	Operating temperature	℃	−20℃ ~ +80℃
Lag	≤%F·S	± 0.03	Input resistance	Ω	750 ± 20 Ω
Repeatability	≤%F·S	± 0.03	Output resistance	Ω	750 ± 5 Ω
Creep	≤%F·S /30min	± 0.03	Safety overload	≤%F·S	150% F·S
Zero output	≤%F·S	± 1	Insulation resistance	MΩ	≥5000 MΩ
Zero temperature system	≤%F·S /10℃	± 0.03	Recommended excitation	V	5V - 15V

**Table 2:** Technical parameters of the ultrasonic distance sensors.

Measurement Range	50 MM ~ 500 MM
Accuracy	0.30%
Resolution	0.1 mm
Measuring frequency	16 KHz
Frequency of sensor center	200 KHz
Working voltage	10 ~ 30 VDC
Power consumption	20 MA
Operating temperature	−20℃ ~ +70℃
Storage temperature	−40℃ ~ + 80℃
Housing material	Nickel-Plated brass
Weight	165 g
Wire length	2 m

**Fig 4:**
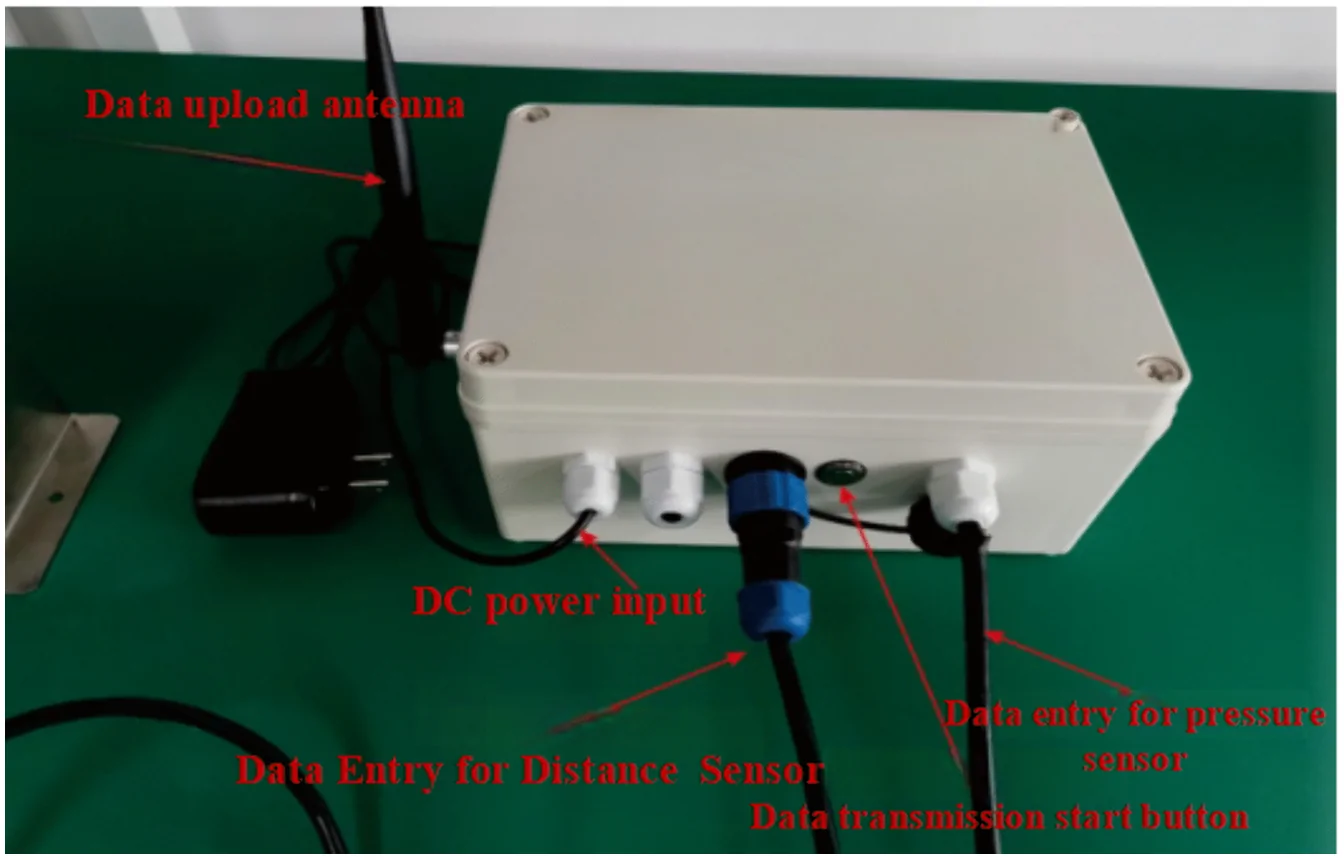
Data acquisition terminal (Republished from Shenzhen Antai Data Monitoring Technology Co., Ltd. under a CC BY license, with permission from Shenzhen Antai Data Monitoring Technology Co., Ltd., original copyright 2020).

**Fig 5:**
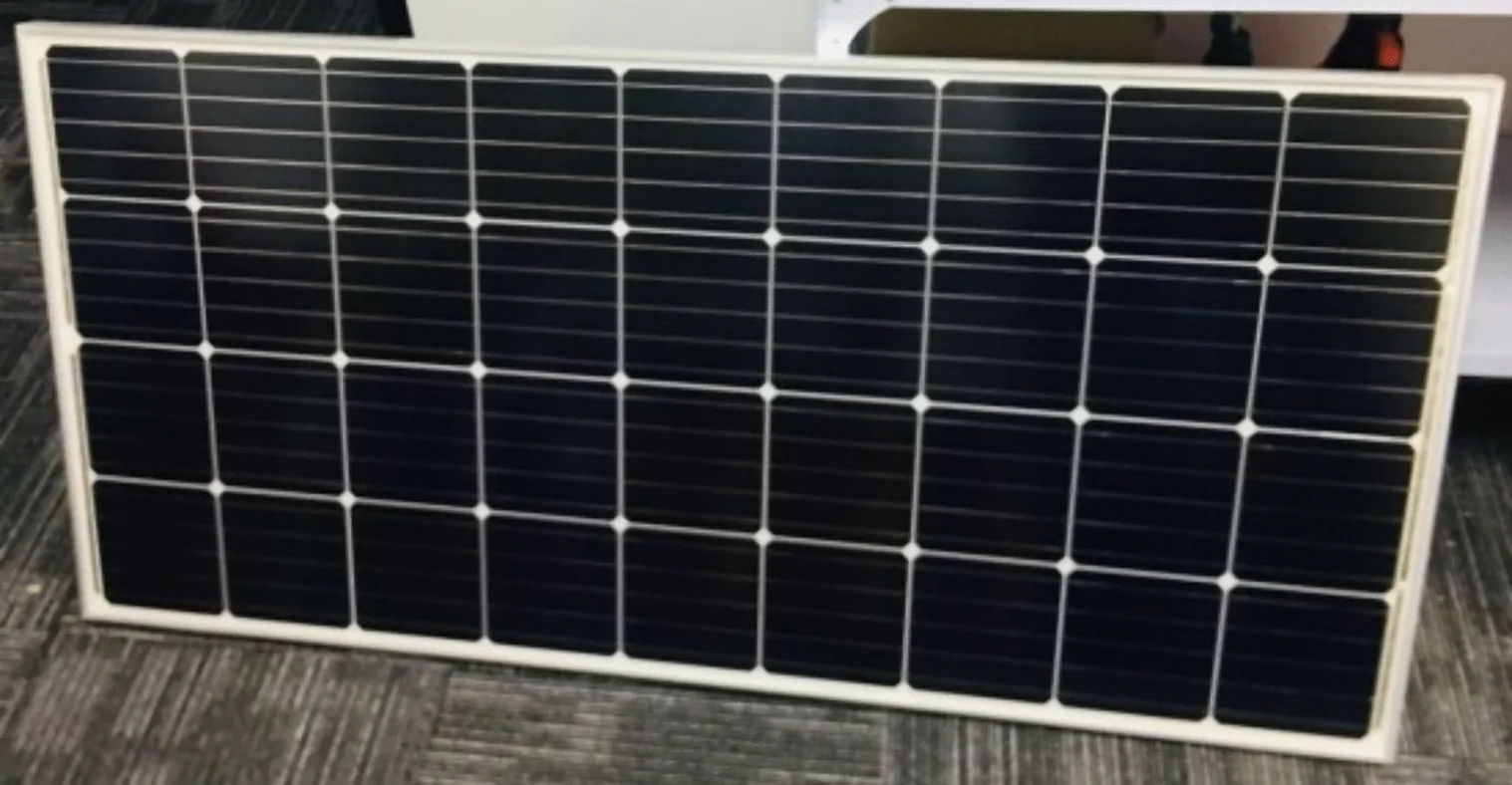
Solar panel (Republished from Shenzhen Antai Data Monitoring Technology Co., Ltd. under a CC BY license, with permission from Shenzhen Antai Data Monitoring Technology Co., Ltd., original copyright 2020).

### Cloud data processing system and typical monitoring results

Once the gateway collects the data, it is relayed to the cloud computing tier. The cloud server is designed to process various data formats, including structured data (e.g., distance, impact pressure, and temperature), semi-structured data (e.g., file formats), and unstructured data (e.g., images, and video streams). All these data types are stored in the database of cloud computing layer before being directed to various display modules across web and mobile applications. For example, pressure sensor data is visualized as statistical graphs in the web-based data display section, while on-site monitoring images are presented in the visualization module of both platforms.

The cloud computing server utilizes the MySQL database service to store historical big data. As a result, the vast quantity of historical data does not consume the local storage space. Additionally, warning signal determination occurs in the cloud computing layer. The warning signal is decided according to the threshold value set for each monitoring parameter. When a warning is triggered, the signal will be prominently displayed on the web. Moreover, it will be sent to users via push notifications in the mobile application and text messages.

Regarding the data-processing module, one of the most critical issues is the false alarm which can occur easily due to various reasons. As shown in Fig 6(a), if the results exceed the trigger level by five times, the triggering will be considered a real trigger. For the false trigger as shown in Fig 6(b), the false alarm value will be brought back to normal using trend analysis. In the system, temperature is a factor which can affect the results, and a temperature adjustment is required. The effect of sensor readings with respect to temperature is usually provided by the manufacturer, and such calibration will be implemented in the server system.

**Fig 6:**
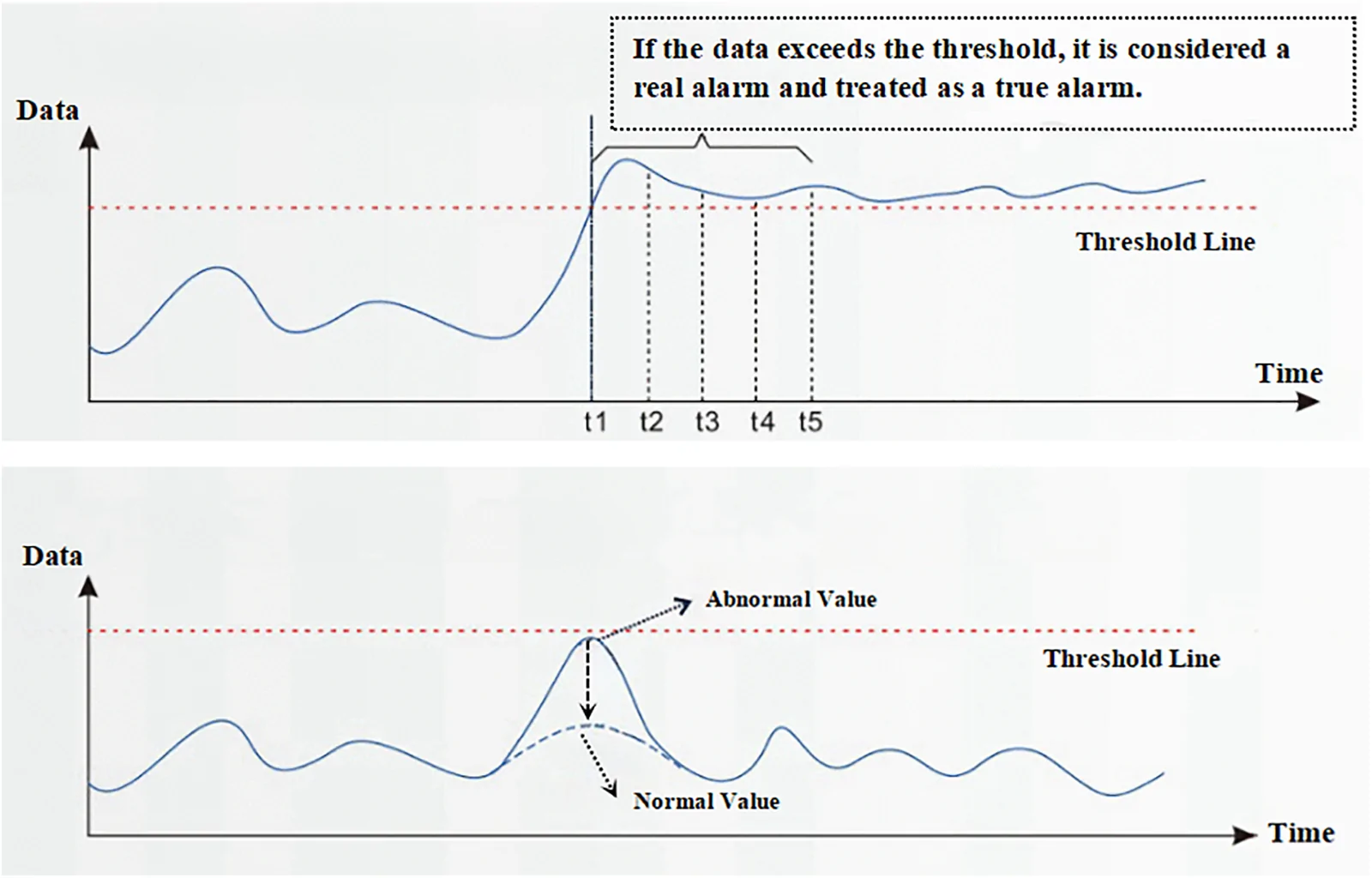
Schematic diagram of real alarm and error warning adjustment. (a) Real alarm. (b) Abnormal data within the threshold is corrected to the normal value (averaged value).

Typical automatic results for the pressure sensors, distance sensors, temperature sensors, and battery sensors are presented in Figs 7–10. Meanwhile, typical images recorded by the industrial camera are shown in Fig 11. These results are considered fundamental to typical rigid barrier monitoring, while rainfall gauges, wind speed, and others can also be included if necessary. These results can be automatically output at regular time intervals or within a given period as specified by the engineers via a web-based system. One important aspect of the present system is that the trigger levels for all sensors can be remotely adjusted by the authorized engineers. An authorization code is issued to all qualified personnel for the adjustment of the trigger levels in this project. Overall, these sensing devices operated normally. The few minor fluctuations are common issues during the operation of the sensors. The minor fluctuations in the sensor readings may be affected by the surrounding environment, such as occasional vibrations, temperature, and humidity. In addition, according to the readings of the temperature sensor (Fig 9), the temperature at this site is relatively stable, remaining between 25 and 35 degrees. Meanwhile, as can be noted from the fluctuations in the battery power (Fig 10), the solar energy and storage battery in this project can coordinate well the power consumption. For example, when the battery power drops to below 70%, the solar panel can immediately replenish the power.

**Fig 7:**
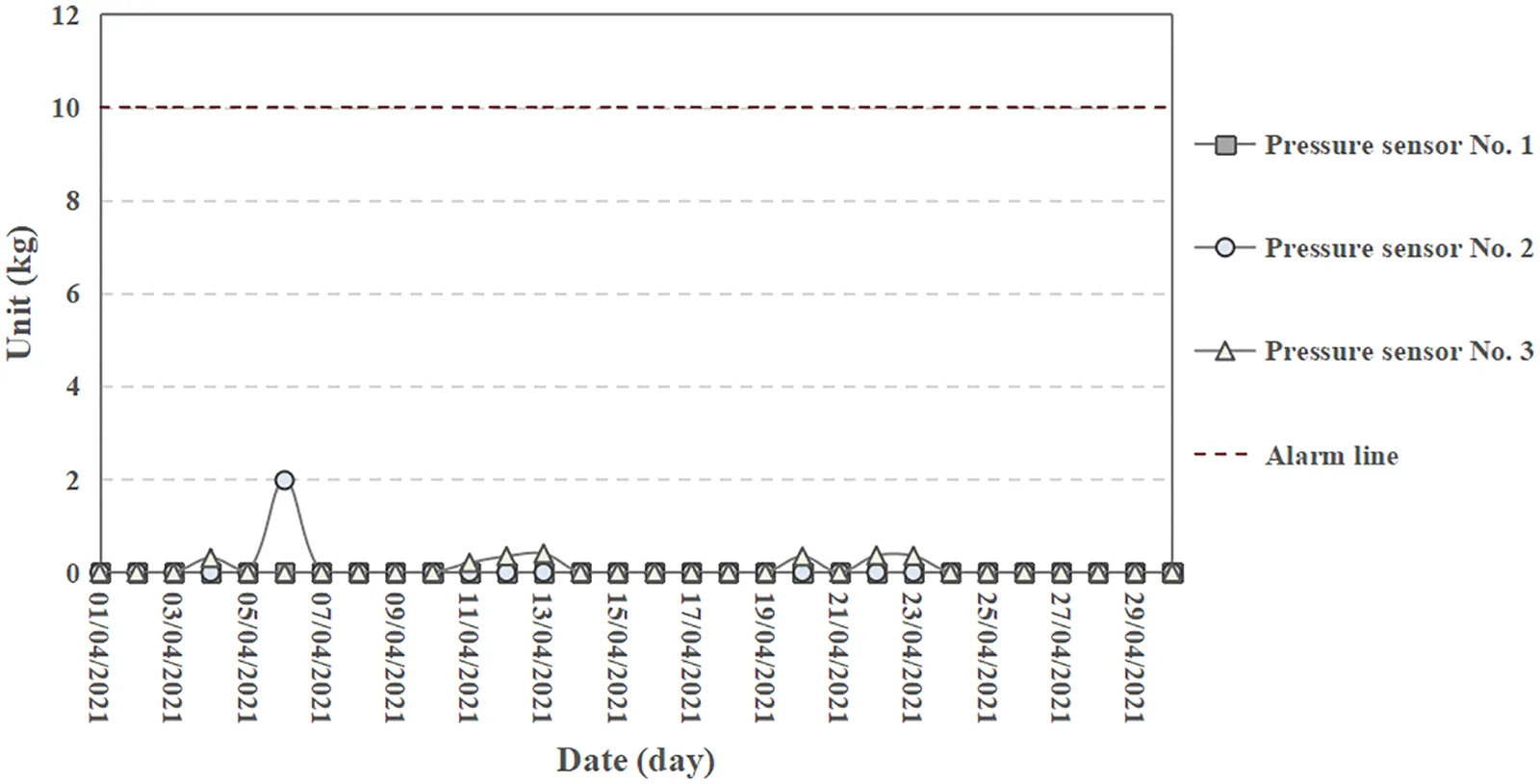
Typical monitoring results recorded by the pressure sensors.

**Fig 8:**
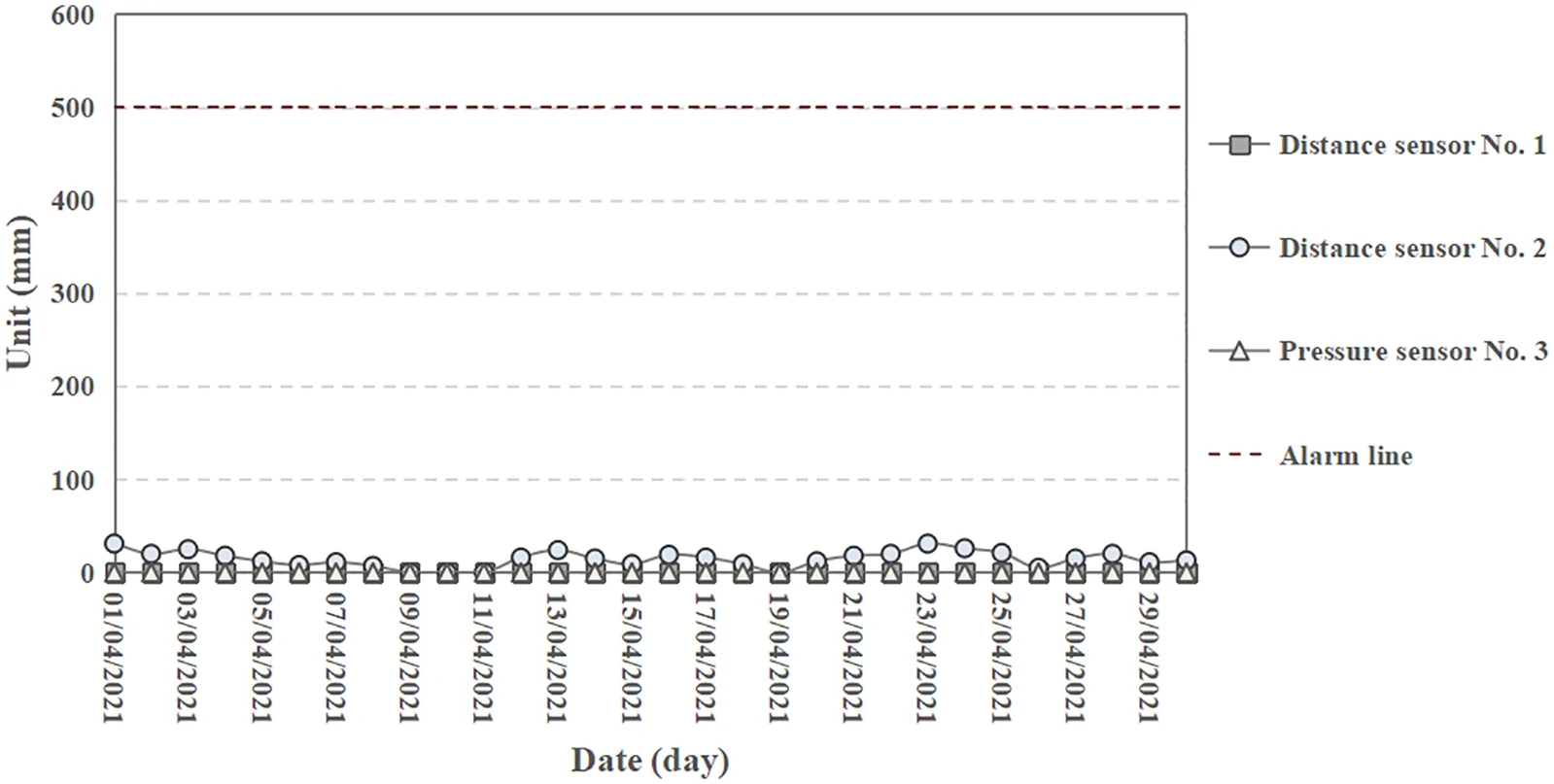
Typical monitoring results recorded by the distance sensors.

**Fig 9:**
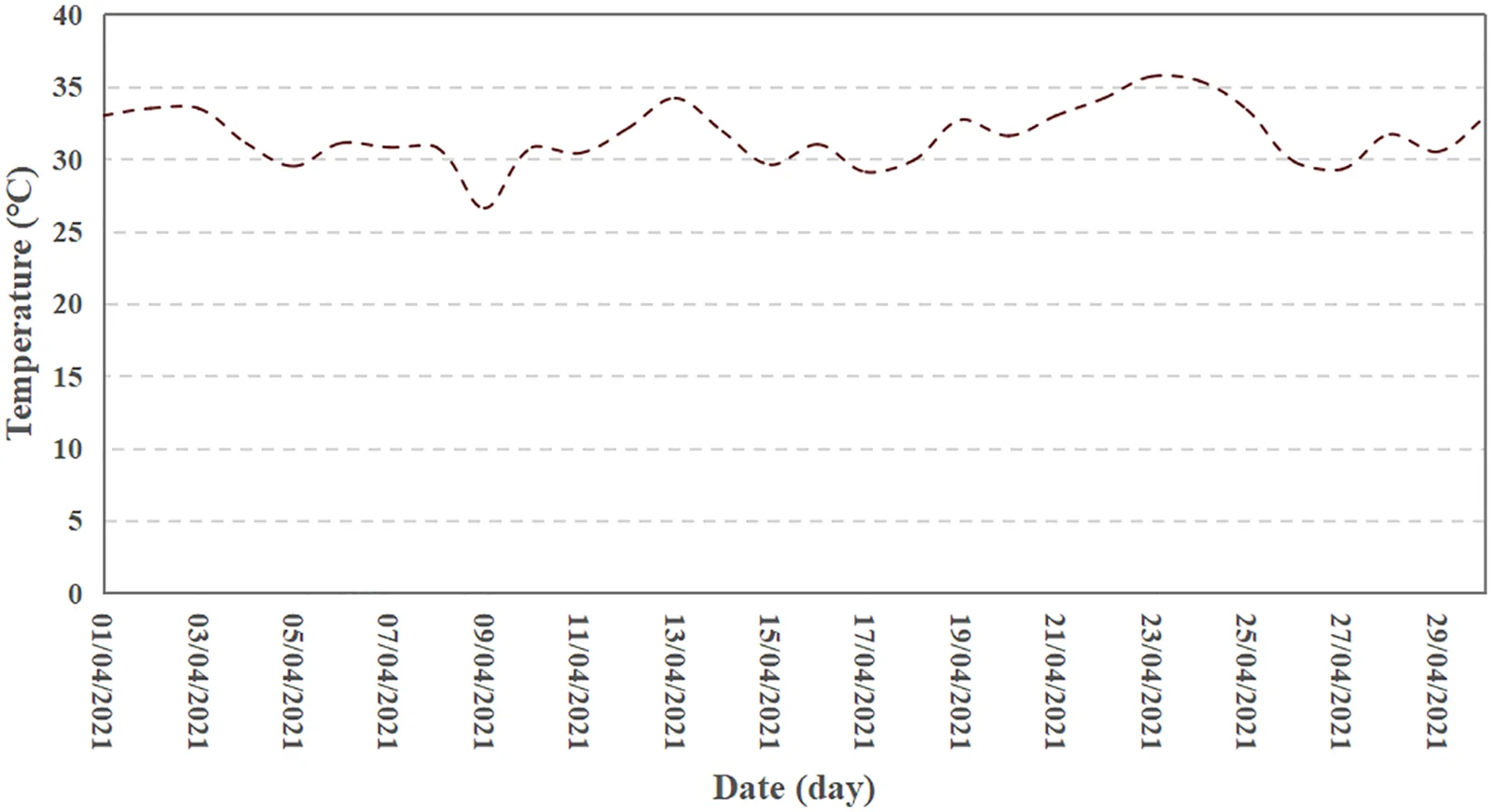
Temperature variation over time.

**Fig 10:**
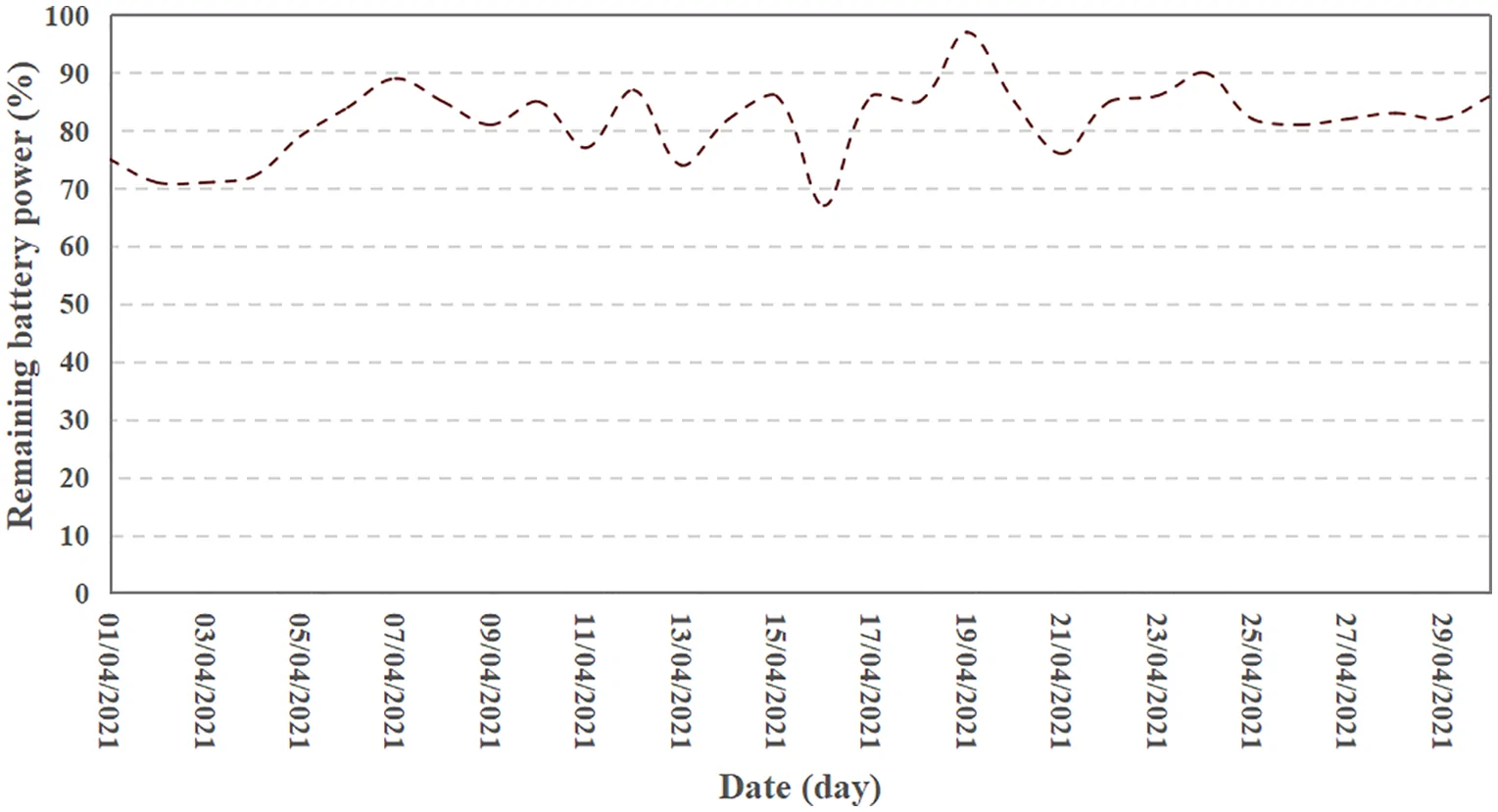
Battery charge over time.

**Fig 11:**
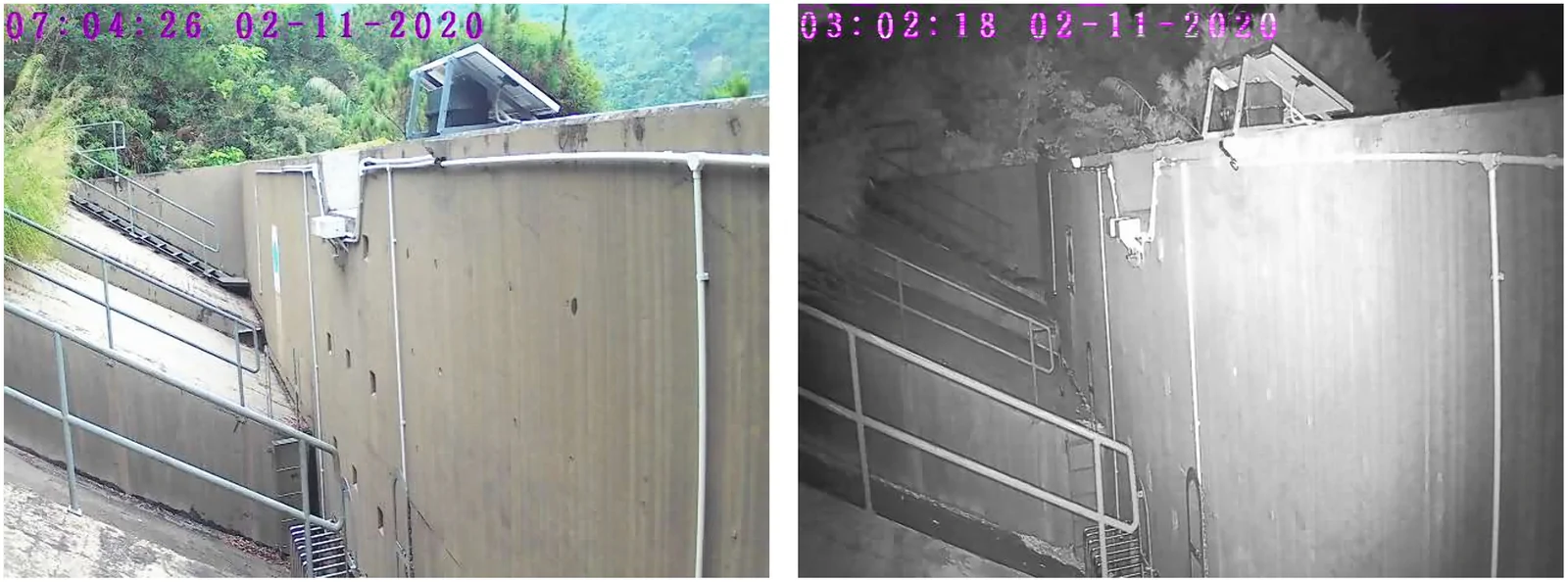
Typical images recorded by the industrial camera:; and (b) image at night (Republished from Shenzhen Antai Data Monitoring Technology Co., Ltd. under a CC BY license, with permission from Shenzhen Antai Data Monitoring Technology Co., Ltd., original copyright 2020).

Furthermore, the stable baseline of pressure and distance monitoring data (Figs 7 and 8) throughout the cycle confirms no debris flow initiation or continuous impact load on the rigid barrier, which is consistent with on-site investigation results. The minor fluctuations in readings, apart from environmental interference, also reflect the micro-deformation of the rigid barrier and slope surface sediment dynamics under daily rainfall erosion, demonstrating the ability of the system to capture potential precursor signals of debris flow. Meanwhile, the stable temperature range (Fig 9) ensures consistent measurement accuracy of sensing elements, avoiding data deviation caused by thermal deformation of the barrier and sensor components, which is critical for reliable identification of debris flow impact signals. The stable power supply performance (Fig 10) verifies that the solar-battery collaborative system fully meets the long-term field operation requirements of the system, which is the fundamental guarantee for continuous monitoring of sudden debris flow events.

### Design paradigm and scientific implications of the IoT monitoring system for debris flows

Fundamentally, the design of this IoT monitoring system is deeply integrated with the structural characteristics of rigid debris flow barriers, realizing the organic combination of disaster prevention structures and monitoring equipment that aligns with the developmental traits of debris flow disasters. This innovative system architecture for debris flows IoT monitoring, which is closely coupled with rigid barrier structures, has not been systematically elaborated in previous studies. In particular, the dual-mode monitoring scheme (surveillance mode and detection mode) is formulated targeting the sudden onset of debris flows, which balances efficient capture of potential disaster signals and low-consumption long-term operation of field equipment. Furthermore, the collaborative configuration of pressure sensors, distance sensors and industrial cameras matches the multi-dimensional monitoring needs of debris flow dynamic processes, forming a complete design logic for perceiving debris flow kinematic features. Herein, distance sensors are used to monitor the distance between the debris flow mass and the rigid barrier in a real-time manner, providing early warning before impact. Pressure sensors are adopted to record the impact load when debris flows hit the barrier, which is critical for structural safety evaluation and theoretical research. Industrial cameras work in coordination with the above two types of sensors, and will be synchronously triggered to switch to the high-frequency shooting mode when the system enters the detection mode, so as to record the dynamic trajectory and morphological characteristics of debris flows in real time and provide complete visual verification for the quantitative data collected by the distance and pressure sensors. Although rainfall is an important triggering factor, the primary goal of this debris flow monitoring system is to capture the movement and impact process of the disaster body. Rainfall data can be integrated into the cloud platform as auxiliary information in future applications, but it is not the core perception unit for directly identifying debris flow initiation and impact behavior.

Although no debris flow disaster occurred at the project site during the whole monitoring cycle, the complete monitoring data obtained fully demonstrates that all functional modules of the system maintained stable and normal operation in the field, verifying the operability and functional integrity of the proposed system. Ultimately, the design concepts of solar power supply, remote threshold adjustment and data redundancy not only guarantee the long-term stable operation of the system in harsh field environments, but also establish a scientific design paradigm for the engineering application of debris flow IoT monitoring.

### Cases 2 and 3: IoT monitoring systems for landslides

The framework of the IoT monitoring system for landslides is also composed of the acquisition end and cloud data processing systems, as shown in Fig 2. In an IoT monitoring system for landslides, the data acquisition end typically includes sensors related to structural displacement, rain gauges, etc. Additionally, the power supply system employs a combination of solar energy and batteries to ensure long-term operation in environments without sunlight, such as rainy days and nights. Furthermore, the industrial camera is often integrated into the system. The purpose of installing video surveillance equipment is to allow users to verify the authenticity of data and signals through real-time site images transmitted by the cameras when abnormal monitoring data or error warning signals occur. Sensor monitoring data and video images are collected by the data acquisition gateway and transmitted via a wireless transmission network to the cloud. The monitoring data will be processed in the cloud, including filtering out abnormal data and determining warning signals. Here, the configuration for the cloud data processing system is the same as that described in the previous section.

The IoT landslide monitoring system adopted in this study follows a mature three-level early warning scheme that has been verified in engineering applications [21], with reproducible warning thresholds, decision criteria and operational response protocols formulated in strict accordance with the *Technical Code for Building Slope Engineering (GB 50330−2013)* [32] issued by the Ministry of Housing and Urban-Rural Development of China. For the core early warning indicator of cumulative displacement of the slope, the red warning threshold is set as the smaller value between *H*/500 and 20 mm, where *H* is the slope height, with the auxiliary rate threshold of continuous 3-day displacement velocity ≥ 2 mm/d; the yellow warning threshold is set as the smaller value between *H*/600 and 17 mm, with the auxiliary rate threshold of single-day displacement velocity ≥ 2 mm/d. In addition, the early warning decision adopts a dual-control criterion. Here, the core indicator is the deep displacement of the slope. The auxiliary check indicators are surface inclination, rainfall and image data. These aim to avoid false alarms caused by single-parameter abnormal fluctuations. Corresponding to the three-level warning mechanism (green for safety, yellow for attention, and red for danger), the automatic monitoring system implements an adaptive monitoring frequency adjustment strategy: the monitoring frequency is once every 2 hours under the green safety state, increased to once every 1 hour under the yellow warning state, and up to once every 5 minutes under the red warning state.

The warning thresholds used in Cases 2 and 3 are adopted from the cited Chinese technical code and prior engineering practice and are applied here to the investigated slopes; they should not be interpreted as universally applicable to all regions or slope types. For deployment in other settings, threshold values should be calibrated according to local soil and rock properties, slope geometry and gradient, groundwater and rainfall characteristics, expected failure mode, sensor accuracy, and applicable regional regulations. The present cases therefore validate the operational implementation of the threshold-based warning logic rather than the universal validity of the numerical thresholds.

The standardized operational response protocols matching the warning levels are established simultaneously. When the yellow warning is triggered, the system automatically increases the monitoring frequency, pushes the early warning information to the local competent authority and on-site management party, and strengthens the on-site inspection. When the red warning is triggered, the system immediately sends the formal warning letter to the competent authority, project owner and surrounding residents. It also organizes experts to conduct on-site verification, initiates the emergency evacuation plan for personnel, and carries out emergency reinforcement of the slope.

In this section, two representative IoT landslide monitoring cases will be presented to illustrate the practical applications of such systems. Here, in both Cases 2 and 3, early warnings for landslides induced by heavy rainfall are successfully triggered by the IoT system. It should be noted that no permits were required for the field monitoring work in this study. The research areas are not protected areas for wild animals and plants. They are also not regions where scientific research is prohibited by government agencies. In addition, this study focuses on slope disaster prevention and serves livelihoods for residents. It does not involve any restricted operations or protected ecological resources.

### Case 2: IoT monitoring system for a retaining wall-reinforced slope with successful early warning

Case 2 is a landslide project located in the Baigong Ao industrial zone, Shenzhen, China. The overview of the site is shown in Fig 12. Here, the total length of the slope is about 150 m, and the height is from 3 m to 20 m. Meanwhile, the slope angle is from 60° to 70°. The slope is formed by manual excavation, and the masonry retaining wall is adopted as the supporting structure, which is divided into three steps. Each step of the retaining wall is from 3 to 5 m high, and the total height of the retaining wall is about 12 m. The trees on the slope are toppling, which have collapsed in history. Meanwhile, the toe of the slope is about 3 m away from the residential building

**Fig 12:**
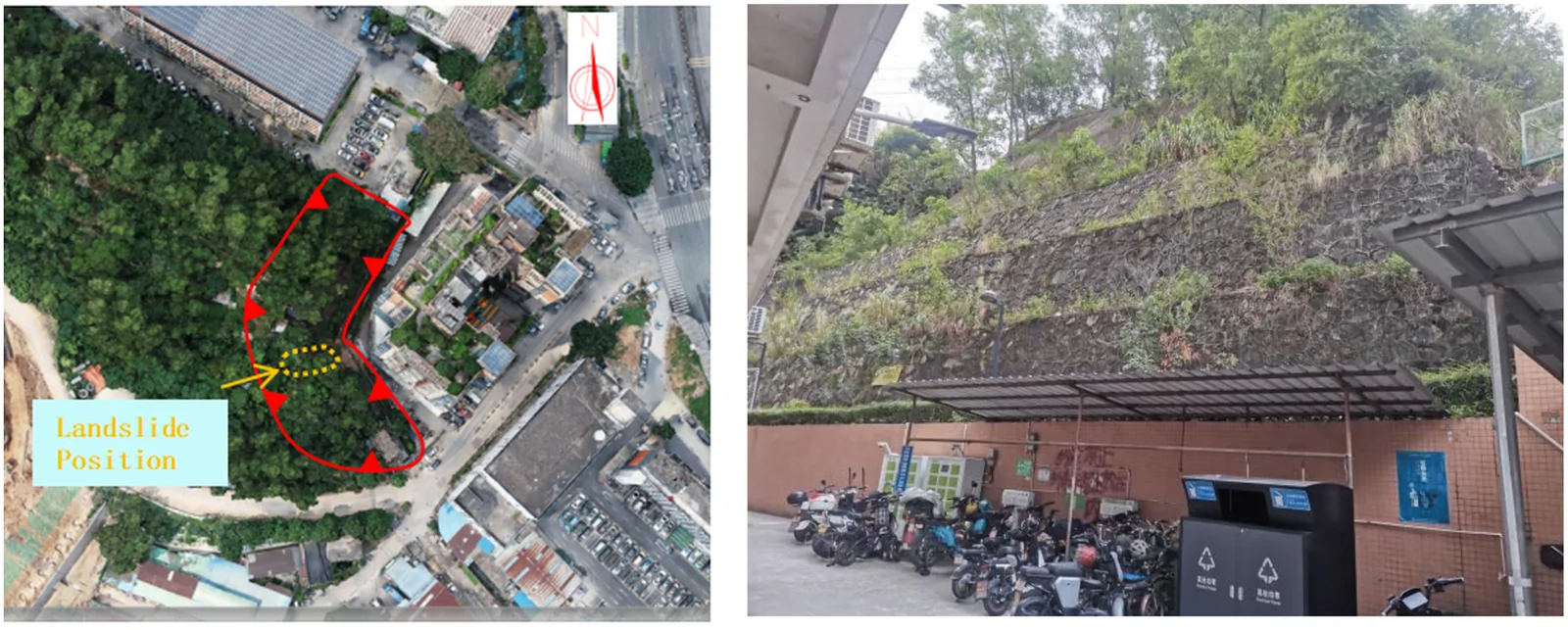
Overview of the slope in Case 2 (Republished from Shenzhen Antai Data Monitoring Technology Co., Ltd. under a CC BY license, with permission from Shenzhen Antai Data Monitoring Technology Co., Ltd., original copyright 2023). (a) Plan view showing the monitored slope and landslide position. (b) Elevation view of the slope.

The acquisition end system for this project is shown in Fig 13. In the acquisition end, the displacement of the slope is collected by inclinometer sensors, which are installed in the slope body to detect deep-seated slope displacement. In the meantime, tilt sensors are installed on the retaining wall to detect the movement of the structure. A rain gauge is installed near the site in a tree-free location to accurately monitor the actual precipitation over the monitored area. Moreover, in this acquisition end system, an industrial camera is installed on the rooftop of a nearby building to ensure the camera can monitor the site environment from an elevated position while being powered by the mains electricity supply. It should be noted that in this project, both the rain gauge and industrial camera transmit data to the cloud via built-in SIM cards independently, whereas monitoring data from displacement sensors is centralized at the data gateway for transmission.

**Fig 13:**
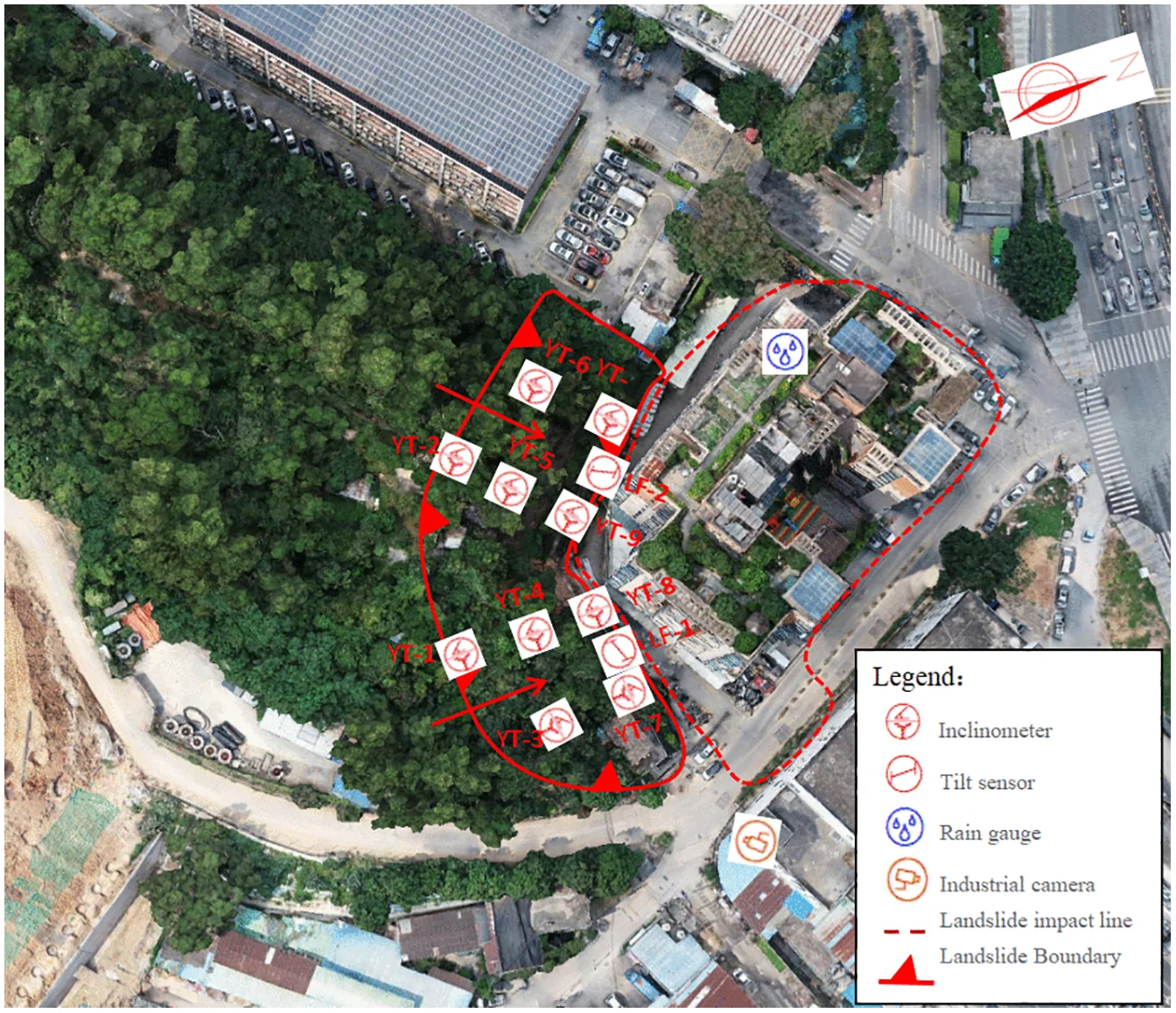
Overview of the acquisition end system in Case 2 (Republished from Shenzhen Antai Data Monitoring Technology Co., Ltd. under a CC BY license, with permission from Shenzhen Antai Data Monitoring Technology Co., Ltd., original copyright 2023).

The typical monitoring data of this case are shown in Fig 14. As can be noted in Fig 14, from September 1st to September 7th, 2023, the cumulative horizontal displacement of YT-1 monitoring point was 0.94 mm. On September 8, due to the heavy rainfall of Typhoon Haikui (250 mm rainfall in the study area recorded by the rain gauge), the cumulative change value of the horizontal displacement at YT-1 monitoring point reached 1494 mm at 1:00 pm, as shown in Fig 14, triggering the red signal warning of geological disaster. In the meantime, the camera captured the scene of the landslide accident. At 10:50 am on September 8, engineers arrived on the site for verification. Shallow-depth failure occurred on the upper slope of the retaining wall, and the sliding volume was about 20 m³(Fig 15). The shallow slope failure is mainly due to the rapid saturation of soil under the influence of short-term heavy rainfall, and the increase of pore water pressure on the sliding body and seepage force along the shallow slope.

**Fig 14:**
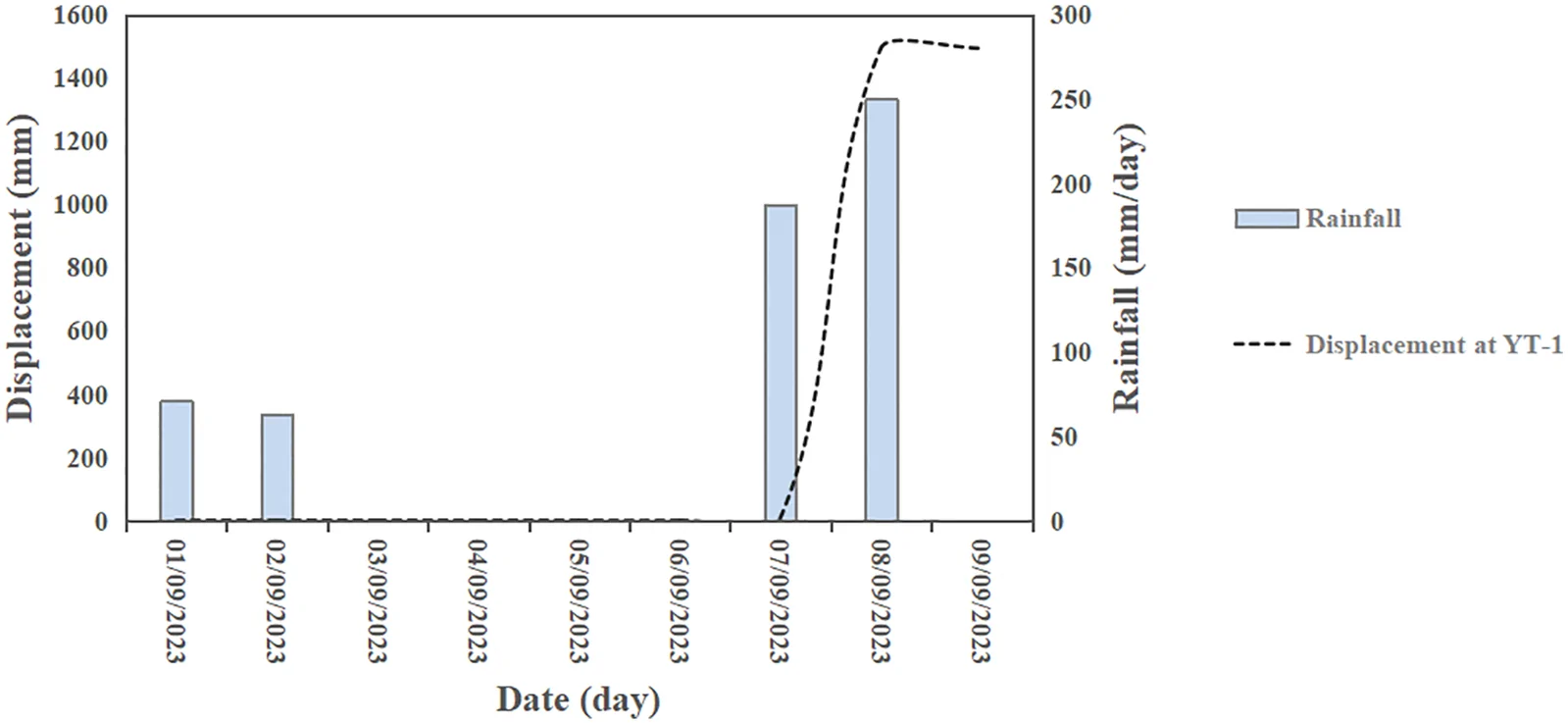
The typical monitoring data in Case 2 from the monitoring point YT-1.

**Fig 15:**
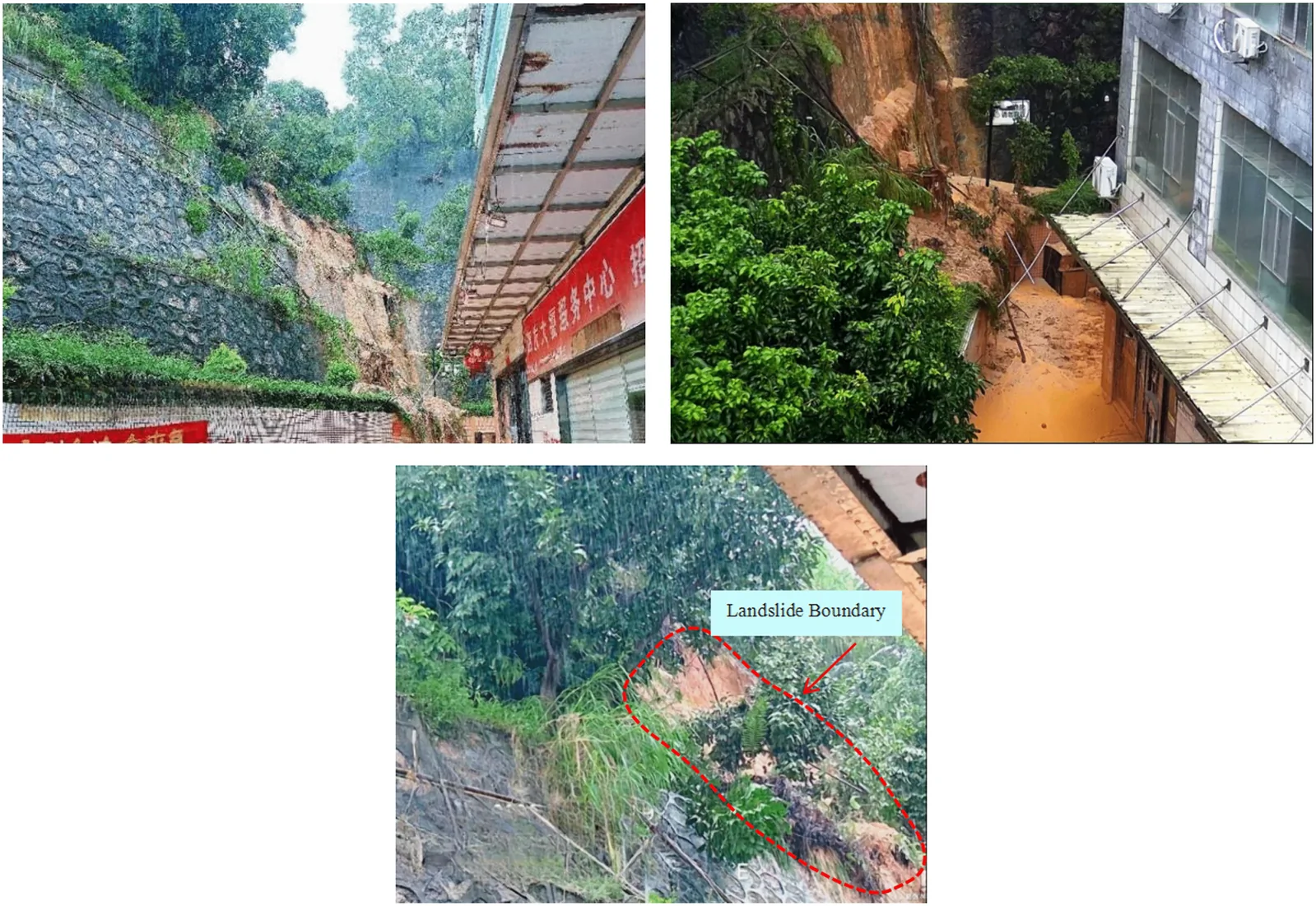
The actual site of slope failure predicted by IoT monitoring with advance warning in Case 2 (Republished from Shenzhen Antai Data Monitoring Technology Co., Ltd. under a CC BY license, with permission from Shenzhen Antai Data Monitoring Technology Co., Ltd., original copyright 2023). (a) Photograph of the site on September 8, 2023. (b) Photograph of the site on September 8, 2023. (c) Failure scar.

Additionally, the monitoring data in Fig 14 clearly reveals the evolutionary mechanism of rainfall-induced shallow landslides. Here, the cumulative displacement remained at a low level in the early stage of continuous rainfall, corresponding to the gradual saturation process of the slope soil. When the cumulative rainfall exceeded the soil saturation threshold, the pore water pressure rose sharply, while the effective stress and shear strength of the shallow soil decreased rapidly, which directly led to the abrupt increase of slope displacement and triggered the red early warning. The synchronous change trend of rainfall and displacement fully verifies that the short-term heavy rainfall of Typhoon Haikui is the dominant inducing factor of this shallow slope failure, and the system can accurately capture the critical transition process of the slope from stable state to instability.

Overall, there was no instability damage to the retaining wall, no damage to the building, and no casualties. With the advanced warning predicted by the IoT monitoring system, there was no major disaster impact.

### Case 3: IoT monitoring system for a semi-reinforced slope with successful early warning

The slope in Case 3 is located on the northeast side of Ruyi Road in Shenzhen, China, which is divided into two parts, as shown in Fig 16. Here, the left part is about 120 m long, and 15 m – 20 m high with a slope angle of 60° - 70°. The slope is a second-grade slope, and the first grade of the slope is supported by masonry blocks. The width of the first grade is about 1.5 m, where a drainage ditch is dug but blocked by various materials. The top of the secondary slope is a natural soil slope with dense vegetation. Some trees have toppled, while the soil on the slope is loose. Besides, small collapsed deposits are noticed at the lower part of the slope. On the right side, the slope length is about 35 m with the height being 5 m – 15 m and the slope angle being from 60° to 70°. There is no stabilization measures on the surface of the slope. A tilted vegetation phenomenon can be seen at the top of the slope, and soil erosion occurs in some areas of the slope.

**Fig 16:**
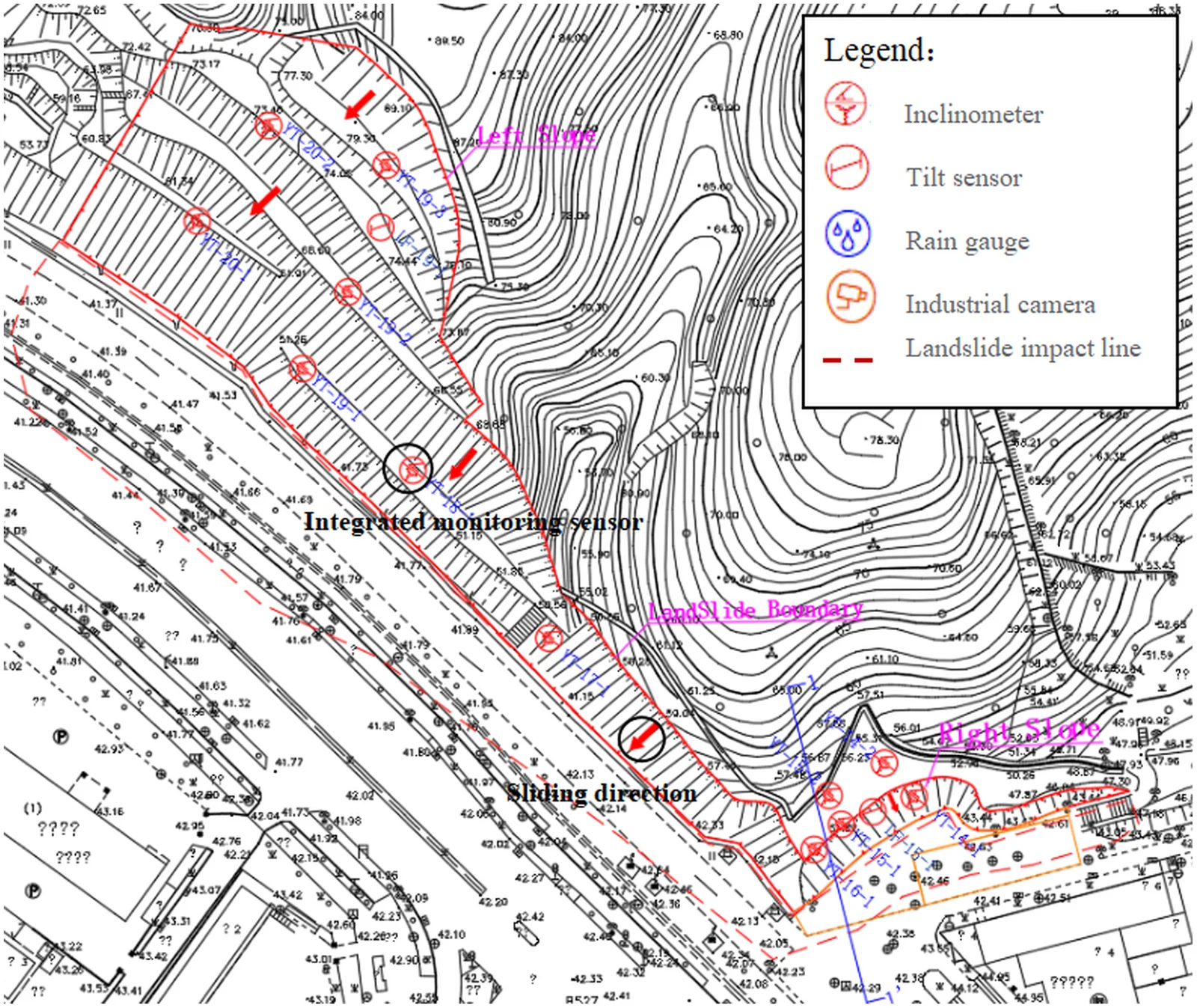
The layout of the monitoring points of Case 3.

The layout of the acquisition end system is shown in Fig 16. Here, a number of inclinometers are installed in the slope body to detect the deep-seated displacement of the slope. Meanwhile, tilt sensors are installed on the masonry wall to detect the surface movement of the slope. The system is also powered by solar cells and batteries to ensure the long-term operation in environments without sunlight.

The typical monitoring data are presented in Fig 17, which shows the variation of the deep-seated displacement at monitoring point TY-15–1. As can be observed in Fig 17, from August 15 to September 6, the cumulative horizontal displacements at YT-15–1 were negligible. However, as can be noted in Fig 17, on September 7, due to the heavy rainfall of Typhoon *Haikui* (400 mm rainfall in the study area recorded by the rain gauge), at 23:00 on September 7, the cumulative change value of horizontal displacement in the X-axis of YT-15–1 point reached 51 mm, and the cumulative change value of horizontal displacement in the Y-axis reached 22 mm, triggering a red warning signal. At 09:00 on September 8, engineers arrived on the site for verification. It was found that a shallow landslide occurred, and the collapsed soil piled up at the toe of the slope, as shown in Fig 18. No casualties or property losses were caused at the site due to the advanced warning.

**Fig 17:**
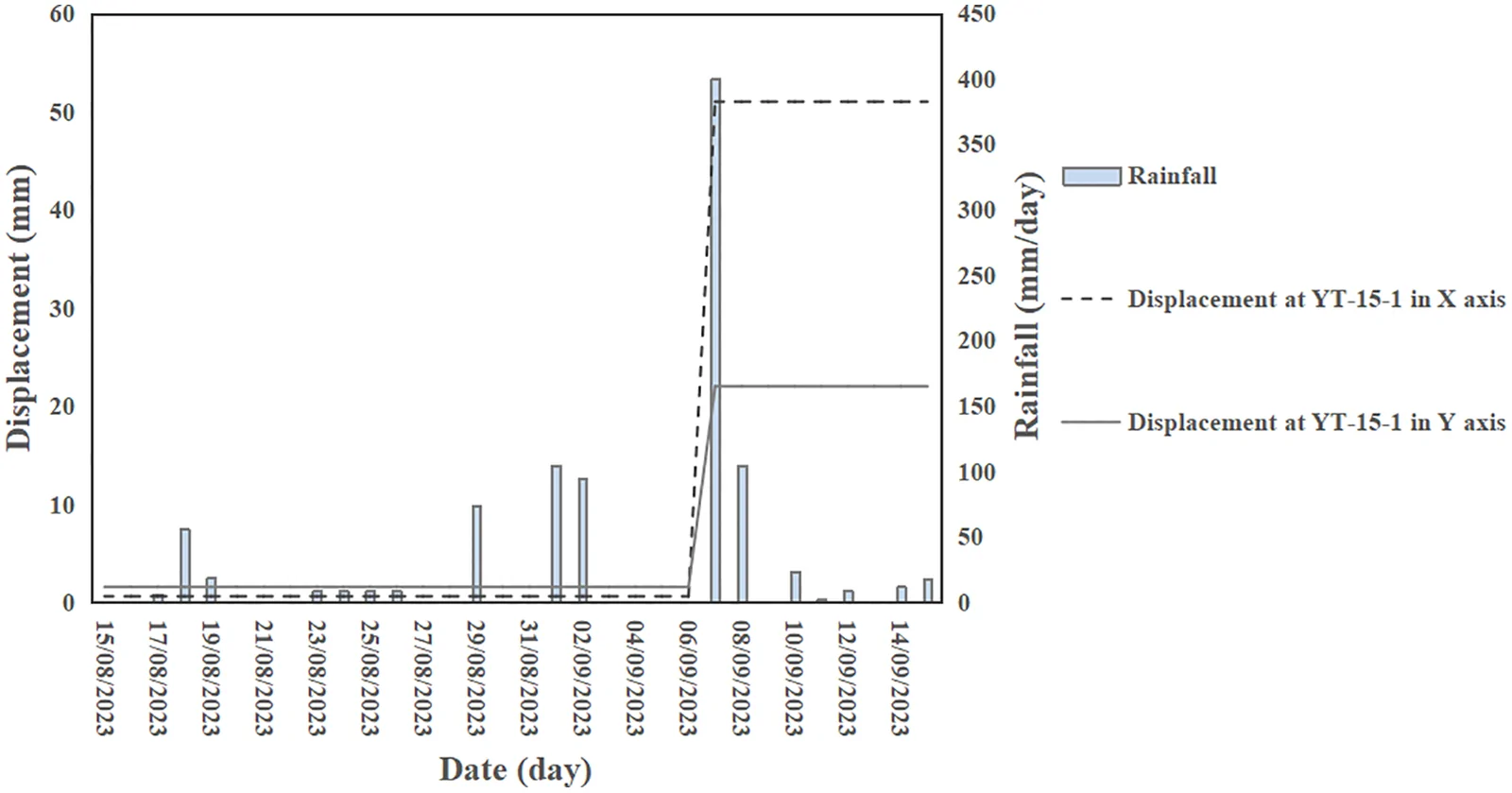
The typical monitoring data in Case 3 from the monitoring point YT-15–1.

**Fig 18:**
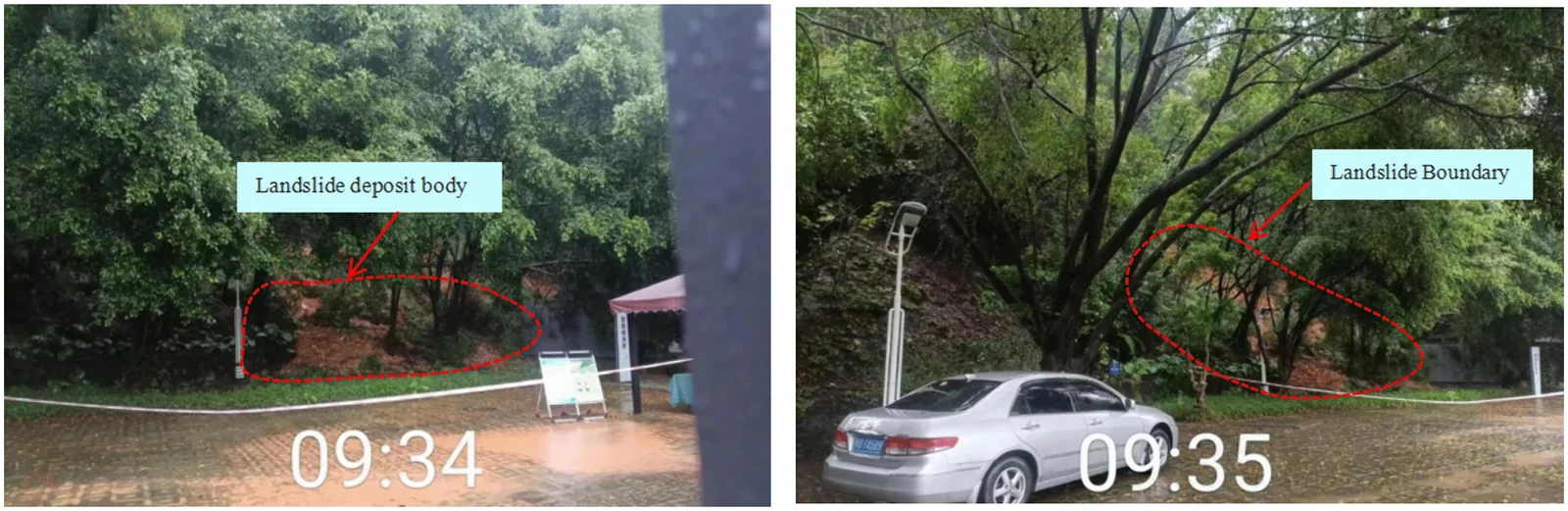
The actual site of slope failure predicted by IoT monitoring with advance warning in Case 3 (Republished from Shenzhen Antai Data Monitoring Technology Co., Ltd. under a CC BY license, with permission from Shenzhen Antai Data Monitoring Technology Co., Ltd., original copyright 2023). (a) Sliding mass accumulated at the foot of the slope on September 7, 2023. (b) Area of the local failure surface.

The stepwise growth of horizontal displacement shown in Fig 17 corresponds to the progressive failure mechanism of the shallow loose soil slope under extreme rainfall. Here, continuous heavy rainfall first weakened the structural plane of the shallow soil, resulting in the initial slow deformation of the slope. With further rainwater infiltration, the seepage force promoted the development of soil cracks and the gradual formation of the sliding surface, leading to accelerated displacement growth and final slope instability. The two-dimensional displacement monitoring data in X and Y directions accurately reflect the movement direction and evolution process of the sliding mass, providing a reliable basis for landslide early warning and on-site verification.

### Design paradigm and scientific implications of the IoT monitoring system for landslides

Overall, the IoT monitoring system for landslides adopts a unified architecture of field acquisition end and cloud data processing, targeting the evolutionary characteristics of rainfall-induced landslide disasters. With the cloud platform as the core linkage hub, the system builds a closed-loop collaborative working mechanism with deformation sensors, rain gauges and video monitoring equipment as the core perception units. Deformation sensors act as the core benchmark for slope stability discrimination, providing quantitative basis for early warning decision-making in strict accordance with the specification threshold. Rain gauges serve as the pre-capture unit for disaster inducing factors, monitoring real-time rainfall parameters to provide inducement verification for slope deformation anomalies. Video monitoring equipment undertakes dual core functions of on-site equipment maintenance and abnormal data verification, which not only monitors field equipment all-weather to avoid man-made damage and other abnormalities, but also remotely verifies the authenticity of abnormal data and warning signals without manual on-site inspection, effectively filtering false alarms caused by equipment faults.

The three types of equipment form a complete collaborative working logic throughout the whole monitoring cycle, covering the full process of disaster inducement capture, slope state perception, and data authenticity verification. The cloud platform dynamically adjusts the monitoring frequency of deformation sensors based on real-time rainfall data captured by rain gauges, enabling targeted capture of potential rainfall-induced deformation signals. When deformation sensors detect abnormal displacement data, the cloud platform first performs coupling analysis between deformation and rainfall data to verify the matching degree between deformation anomalies and rainfall inducement. For abnormal data without clear inducement matching, the system immediately links video monitoring equipment to collect real-time high-definition images, allowing management personnel to remotely check the installation status of equipment and the actual state of the slope. This enables rapid differentiation between real deformation anomalies and false signals without manual on-site verification. This multi-source collaborative configuration meets the multi-parameter monitoring requirements of landslide disaster evolution, forms a reliable perception logic for slope stability state, effectively addresses the issues of single-parameter false alarms and high on-site operation and maintenance costs in remote areas, and ultimately constitutes a core part of the practical design paradigm for landslide IoT monitoring applications.

## Discussion

### Discussion on the system uncertainty, robustness and operational risks

The main sources of uncertainty in the operation of the proposed IoT monitoring system include occasional fluctuations in sensor readings caused by environmental factors such as field vibration, temperature and humidity changes, as well as the risk of data transmission interruption caused by unstable 4G network signals in remote mountainous areas. To mitigate the risks of false alarms and missed warning events, the system adopts a multi-dimensional anti-interference design including multi-sensor data redundancy, trend analysis-based abnormal data correction, and temperature adaptive calibration, which effectively reduces the probability of invalid warnings and signal missing.

The robustness of the system under adverse field conditions is fully guaranteed by the integrated design of waterproof packaging for the equipment, solar-battery hybrid power supply that supports continuous operation for more than 30 days without sunlight, and local data storage with break-point resume transmission function, which has been verified by long-term stable operation in high-temperature, high-humidity and heavy rainfall environments in all the cases.

To further improve communication reliability at sites where 4G coverage is unstable, future deployments can adopt a hierarchical redundant communication strategy. Where cellular coverage is available, dual-carrier or multi-network SIM gateways can reduce dependence on a single operator. For low-bandwidth numerical sensor data and critical alarm packets, alternative low-power communication channels, such as NB-IoT where alternative cellular coverage is available, or a local LoRa/LoRaWAN link to a gateway with more reliable backhaul connectivity, can be adopted as auxiliary transmission paths. Because image and video transmission requires substantially higher bandwidth, the primary cellular link can be retained for multimedia data, while critical alarm information is preferentially transmitted through an available backup channel. The existing local SD-card storage can then serve as the final data-recovery layer, thereby minimizing the risk of permanent data loss during temporary communication failures.

Only a small number of minor operational anomalies occurred during the full monitoring cycle of the three engineering cases in this study, including short-term signal fluctuations under extreme rainfall conditions, slight reading drift caused by long-term field environmental changes, and individual temporary sensor operation anomalies. These anomalies were effectively resolved eventually, without causing any missed warning events, system failures or adverse impacts on on-site disaster prevention, while the long-term operational performance of the system in more complex and diverse geological environments may be further verified through the accumulation of more engineering cases in future research.

### Discussion on differentiation between standard IoT practices and the proposed technical schemes

Standard IoT monitoring practices generally follow a universal three-tier architecture comprising a sensing terminal, data transmission link, and cloud platform, with the core focus on realizing the basic functions of data collection, wireless transmission, and centralized storage. Such practices mostly adopt general-purpose sensing devices, fixed data acquisition frequencies, and single-threshold warning logic, which are essentially scenario-based transplantation of general IoT technology, lacking targeted adaptation to the unique characteristics of slope hazard monitoring.

Compared with standard IoT practices, this study proposes three core differentiated innovations specifically for slope hazard monitoring. In terms of novel technical adaptations, we break the independent design mode of conventional IoT systems and engineering protection structures, propose an innovative debris flow IoT monitoring scheme deeply integrated with rigid barrier structures, customize a multi-dimensional collaborative sensing system matching the impact dynamic characteristics of debris flows, and design a dual-mode operation strategy balancing efficient disaster signal capture and long-term low-power operation of field equipment. For field-validated configurations, we construct a multi-source collaborative early warning configuration for rainfall-induced landslides, and verify the reliability of a series of field-adapted configurations through three engineering cases and two successful early warning events. For generalized design principles, we extract a unified IoT architecture applicable to both debris flow and landslide monitoring, systematically integrate full-chain technical modules, and clarify the general architecture criteria and disaster-specific differentiated design rules, forming a replicable universal design framework dedicated to slope hazard IoT monitoring.

### Discussion on the advantages and disadvantages of IoT monitoring

For slope engineering, it is well known that there are many limitations in the current practice. In particular, there are various uncertainties associated with the debris flow and landslide problems in the stability evaluation and design process. Thus, in addition to the adequate design, the monitoring and issuance of warnings are also important.

The integration of IoT monitoring systems for debris flow and landslide prevention, as demonstrated in the three case studies, highlights significant advancements over traditionally manual monitoring methods. IoT systems offer unparalleled advantages in real-time data acquisition, automated response mechanisms, and multi-parameter integration, which are critical for mitigating the risks associated with slope failures. Through the collaborative operation of pressure, distance, tilt, and rain sensors alongside industrial cameras, IoT systems can collect and transmit data to the cloud in real time, enabling instant alerts. For example, the red warnings were triggered during Typhoon Haikui in Cases 2 and 3, which substantially reduced delays inherent in manual inspections. Meanwhile, the fusion of multi-parameter analyses (e.g., impact pressure, displacement changes, and visual records) provides a comprehensive assessment of hazard dynamics, while the solar-powered system ensures long-term and low-cost operation in remote or hazardous areas. Additionally, IoT systems effectively reduce risks associated with on-site human operations through automated alarms and remote monitoring (Cases 2 and 3), successfully preventing casualties in multiple disasters and highlighting their dual improvements in safety and efficiency.

Despite the significant advantages of IoT monitoring, limitations warrant attention. The deployment costs in the initial stage are usually high, which include investment costs for installing and commissioning sensors, gateways, and solar facilities. Moreover, the long-term on-site operation and maintenance entail certain technical and labor thresholds, and equipment inspection, calibration, and replacement in remote mountainous areas are relatively inconvenient. Besides, technical dependencies pose risks, such as data transmission interruptions due to unstable networks. The system also faces adaptation challenges under extreme geological and meteorological conditions (e.g., severe cold and heavy blizzards), and its operational stability in such harsh environments has not been fully verified. Meanwhile, sensor calibration deviations in the wild environments (e.g., occasional vibrations, temperature, and humidity) may trigger false alarms. For instance, in Case 1, false alerts were corrected via trend analysis, but regular maintenance is still required. However, these issues can be gradually mitigated through technological advancements and standardized maintenance. The present field cases demonstrate operational advantages in continuous data acquisition, remote access, and automated warning; however, the magnitude of improvements relative to traditional manual monitoring in response time, labor input, and cost has not been quantified in this study.

### Discussion on the limitations of the current work

The three engineering cases in this study cover the core scenarios of slope disaster monitoring in practical engineering, including two typical instability mechanisms, common geological conditions and extreme rainfall-triggered events, but limited by the scale of this batch of engineering practice, this work does not establish a broader multi-case comparative dataset or carry out cross-scenario systematic validation, which brings certain limitations to the full demonstration of the general adaptability of the proposed IoT monitoring system.

In addition, the available cases are geographically concentrated in Hong Kong and Shenzhen, and the two landslide cases involve relatively steep slopes with gradients of approximately 60°-70°. They therefore do not represent the full range of soil compositions, slope geometries, climatic conditions, and failure mechanisms encountered in other regions. Future validation should include multi-regional sites with a wider range of slope gradients and geotechnical materials, such as cohesive, granular, residual, and strongly weathered soils, together with different rainfall and groundwater regimes. Such expanded case studies are necessary to evaluate the transferability of the proposed architecture and to determine the degree of site-specific or regional calibration required for warning thresholds and sensor configurations.

While the stable on-site operation and successful early warning practice of the system in the three cases have fully verified its core functional effectiveness, this study has not yet conducted a quantitative performance evaluation of the proposed IoT system covering core operational indicators, nor has it established a parallel control dataset of traditional manual monitoring methods at the same site and monitoring cycle to carry out full-dimensional quantitative comparative analysis of the technical superiority of the system. These supplementary quantitative analyses cannot be retrospectively completed due to objective engineering constraints, as all projects involved have been completed and the on-site monitoring equipment has been dismantled. The case-specific conclusions regarding stable system operation and successful warning events are nevertheless supported by the complete field monitoring records and the two independent Typhoon Haikui-induced landslide cases.

Future validation should therefore adopt a prospective side-by-side comparison at the same slope and over the same monitoring period, with IoT measurements and warning records paired with scheduled manual measurements and inspections using common reference points. The comparison should quantify data latency and warning lead time, labor hours and associated monitoring cost, missing-data rate, false-alarm rate, and consistency of deformation measurements. A same-site, same-period design would allow the practical gains and limitations of the IoT system to be quantified without confounding differences in site conditions or event severity.

## Conclusion

The application of IoT technology in debris flow and landslide monitoring provides an optimized technical path for slope disaster management, and offers real-time and multi-dimensional solutions for slope disaster prevention and control in the engineering scenarios covered by this study. The IoT monitoring systems for debris flows and landslides designed in this study share the core design fundamentals of real-time data acquisition, cloud-based automatic early warning and sustainable solar-battery power supply, while differing in sensor configuration and monitoring focus due to the distinct evolutionary characteristics of the two types of disasters. Specifically, targeting the unique impact dynamics and rigid barrier-based prevention mode of debris flow disasters, this study proposes an innovative IoT monitoring scheme for debris flows that collaboratively integrates rigid debris flow barriers and intelligent monitoring systems, which forms the core targeted and differentiated design of the debris flow monitoring system under the unified architectural framework. This unified architectural framework and core design principles run through the whole design and implementation process of all cases in this study, forming a replicable and standardized implementation logic for slope disaster IoT monitoring.

Based on the system design logic and field application characteristics of the three engineering **cases**, this study systematically integrates full-chain technical modules for the unified IoT architecture, including physico-mechanical sensing devices, a solar-battery collaborative power supply system, video monitoring equipment, data transmission methods, early warning modes and a cloud-based data processing module. The key design principles of IoT slope monitoring systems are summarized accordingly, which ensure the operational stability and anti-interference performance of the system in complex field environments. Also, these principles enable stable adaptability across diverse engineering scenarios including artificial reinforced and semi-natural slopes, laying a technical foundation for accurate identification of slope instability precursors.

The successful early warning cases during Typhoon *Haikui* demonstrate that the proposed IoT monitoring system can capture rapid displacement changes associated with rainfall-induced slope instability and issue automated red warnings in the studied engineering settings. These cases support its practical value for continuous and timely field monitoring; however, quantitative gains in response time, labor cost, and overall monitoring performance relative to traditional manual monitoring remain to be established through parallel controlled comparisons. This study provides a field-verified, practical and implementable technical reference framework for similar slope disaster monitoring engineering practices.

## Supporting information

S1 File S1 Data for Fig 7. S2 Data for Fig 8. S3 Data for Fig 9. S4 Data for Fig 10. S5 Data for Fig 14. S6 Data for Fig 17.
(ZIP)
